# Genistein suppresses the inflammation and GSK-3 pathway in an animal model of spontaneous ovarian cancer

**DOI:** 10.3906/sag-2007-254

**Published:** 2021-06-28

**Authors:** Füsun ERTEN, Engin YENİCE, Cemal ORHAN, Beşir ER, Pınar DEMİREL ÖNER, Patrick Brice DEFO DEEH, Kazım ŞAHİN

**Affiliations:** 1 Division of Biology, Faculty of Science, Fırat University, Elazığ Turkey; 2 Department of Animal Nutrition, Faculty of Agriculture, Ankara University, Ankara Turkey; 3 Department of Animal Nutrition, Faculty of Veterinary Medicine, Fırat University, Elazığ Turkey; 4 Department of Microbiology, Education and Research Hospital, Elazığ Turkey; 5 Department of Animal Biology, Faculty of Science, University of Dschang, Dschang Cameroon

**Keywords:** Genistein, GSK-3, IL-6, TNF-α, laying hens, ovarian cancer

## Abstract

**Background/aim:**

Numerous studies show that cancer risk is reduced by consumption of soy-based foods containing genistein, but its effects on the glycogen synthase kinase-3 pathway (GSK-3) in ovarian cancer is unknown. Therefore, we tested the properties of genistein on inflammatory biomarkers and GSK-3 signaling pathways in the ovaries of old laying hens with ovarian cancer.

**Materials and methods:**

A total of 300 laying hens were distributed into three groups as follows: group 1, animals fed a standard diet (comprising 22.39 mg of genistein/kg of diet); groups 2 and 3, animals fed a standard diet reconstituted with supplementation of 400 mg or 800 mg of genistein/kg of diet, respectively.

**Results:**

Genistein modulated the inflammatory biomarkers by decreasing serum tumor necrosis factor-α (TNF-α), interleukin-6 (IL-6), interleukin-8 (IL-8), and vascular endothelial growth factor (VEGF) compared with control (p < 0.001). Moreover, it upregulated insulin receptor substrate-1 (p-IRS-1) and protein kinase B (p-AKT), but downregulated GSK-3α and β after treatment. It acts in a dose-dependent manner.

**Conclusion:**

Genistein exhibited an anticancer effect by reducing proinflammatory biomarkers levels and inhibiting GSK-3 expression in the ovaries of old laying hens. It is a potential candidate in the chemoprevention and/or treatment of ovarian cancer.

## 1. Introduction

Ovarian cancer has the third highest incidence of gynecological cancer in women globally, and the second most common gynecological cancer mortality [1]. The incidence and mortality of ovarian cancer show remarkable geographic variations at higher rates in industrialized countries. For instance, incidence and mortality in high/very high human development index (HDI) districts are approximately 7.0 and 3.8 per 100,000 women and 5.7 and 4.0 for 100,000 women in low/medium HDI regions, respectively [2]. Lifestyle factors and genetic predisposition are the main risk factors for ovarian cancer development [3]. The etiology of this disease is poorly understood. The key principles are continuous ovulation and gonadotropin overproduction [4]. Malignant transformation may result from overexcitation due to hormonal factors, including estrogen-rich follicular fluid after erosion or extreme gonadotropin levels leading to stimulation via estrogen or estrogen precursors [4]. The high mortality rate of ovarian cancer is linked to difficulties in diagnosis of the early stages of the disease, high rate of recurrence, platinum resistance, and inflammation [5]. Glycogen synthase kinase-3 (GSK-3), an enzyme to regulate glycogen synthesis, has a key role in regulating immune and inflammatory responses by activities of various transcription factors that have been well reported [6].

Inflammation, oxidative stress, and DNA damage are causative factors in carcinogenesis [7]. Ovulation itself has been reported to be related to inflammation at the epithelial and follicle level [8]. In addition, talc exposure, endometriosis, and pelvic inflammatory diseases are other risk factors for developing ovarian cancer, but they do not directly affect ovulation and sex hormone profile [9]. Modern treatment of ovarian cancer is associated with drug resistance, a high risk of recurrence, and severe side effects in some cases [10]. Thus, the investigation for natural compounds with fewer side effects and easy availability is on demand. Studies have shown that high isoflavones content in soy products prevents ovarian cancer [11]. In addition, high soy oil consumption significantly decreased the occurrence of ovarian cancer in women [12]. Isoflavones, a major class of phytoestrogens, are plant-derived compounds present in soy products and other legumes. Genistein (5,7,4’-trihydroxyisoflavone), the most commonly studied natural isoflavone, has been shown to affect the cell cycle of cancer cells progression and apoptosis [13]. In SK-OV-3 cells, genistein prevents cellular proliferation and induces cell cycle hold at the G2/M phase in a dose- and time-dependent manner [14]. In addition, it has been shown that genistein downregulates vascular endothelial growth factor (VEGF) receptors, which are considered critical targets for ovarian cancer treatment. Genistein is compared to other isoflavones, with a potent inhibitory property on VEGF protein secretion [15]. In ovarian cancer cells, high concentrations of genistein induce apoptosis and cell death while low concentrations exhibit antioxidant potential without any cytotoxic effect [16].

To evaluate the effects of genistein in ovarian cancer, laying hens are suitable experimental models because they spontaneously stimulate ovarian cancer largely when stopping egg production similar to women. However, other animals such as mammals and rodents do not spontaneously develop ovarian cancer [17]. In addition, the risk of developing ovarian cancer in chickens as well as in women increases with age and the number of ovulation for life [18]. The high prevalence of ovarian cancer in laying hens depends on age, genetic predisposition, reproductive history, and diet. Due to the similarity between ovarian cancer in laying hens and human ovarian cancer, laying hen is a proper experimental animal model for translational research [19]. This study examined the supplemental genistein properties on inflammatory biomarkers and glycogen synthase kinase-3 signaling pathway in laying hens with ovarian cancer.

## 2. Materials and methods

### 2.1. Animals and design

Three-hundred laying hens (104-week-old; ATAK-S hybrid, *Gallus domesticus* ) were used in this work. The Animal Experimental Ethical Committee of Poultry Research Station (Ankara, Turkey) permitted the study. The birds were divided into three groups (n = 100) and treated as follows: group 1, animals fed a standard diet containing 16.83% crude protein (CP), 11.15 MJ/kg metabolizable energy (ME), and 22.39 mg genistein per kg of diet (Table); groups 2 and 3, birds fed a standard diet reconstituted with the adding of 400 mg or 800 mg of genistein per kg of standard diet at the expense of corn, respectively. The genistein (Bonistein™, 98% aglycone, and 2% starches as a carrier) was provided by DSM Nutritional Products Inc. (İstanbul, Turkey). Animals received 3.01, 52.48, and 106.26 mg genistein/hen per day in the control, low genistein, and high genistein groups, respectively. The birds had food and water ad libitum during the experimental period (78 weeks).

**Table:** The composition of the basal diet1.

Ingredient	g/kg
Corn	625.0
Soybean meal2	274.7
Limestone	75.0
Dicalcium phosphate	15.0
Vitamin-mineral premix3	2.5
Sodium chloride	3.5
Sodium bicarbonate	2.0
Methionine	1.5
Choline chloride	0.8
Chemical analyses, dry matter basis	
Crude protein	168.3
Crude fat	37.1
Crude fiber	35.3
Crude ash	111.0
Calcium	38.0
Phosphorus	3.6
Calculated compositions	
Methionine	5.6
Lysine	9.6
Metabolizable energy, MJ/kg	11.15

1All analyses were conducted in triplicate. Genistein was added to the basal diet at the expense of corn in the amount of 0, 400, and 800 mg per kilogram. After reconstitution of the basal diet, respective experimental diets contained 22.39, 392.82, and 792.72 mg of genistein per kilogram. 2Soybean meal contained the following: 48% protein, 0.75% fat, 5.3% ash, 5.8% fiber, 81.5 mg of genistein/kg, and 68.2 mg of daidzein/kg. 3Supplied per kilogram of diet: retinyl acetate, 12,000 IU; cholecalciferol, 2400 IU; dl-α-tocopheryl acetate, 30 mg; menadione sodium bisulfite, 2,5 mg; thiamine-hydrochloride, 3 mg; riboflavin, 7 mg; niacin, 40 mg; d-pantothenic acid, 8 mg; pyridoxine hydrochloride, 4 mg; vitamin B12, 0,015 mg; vitamin C, 50 mg; folic acid, 1 mg; D-biotin, 0,045 mg; choline chloride, 125 mg; Mn (MnSO4-H2O), 80 mg; Fe (FeSO4-7H2O), 30 mg; Zn (ZnO), 60 mg; Cu (CuSO4-5H2O), 5 mg; Co (CoCl2-6H2O), 0,1 mg; I as KI, 0,4 mg; Se (Na2SeO3), 0,15 mg.

### 2.2. Sample collection

Blood samples and ovary tissues were collected from 10 birds, with ovarian cancer from each group. After centrifugation (3000 × *g* for 10 min), serum samples were collected and stored at –80 °C until further analyses. ELISA (Elx-800, Bio-Tek Instruments Inc, Vermont, USA) determined serum TNF-α, IL-6, IL-8, and VEGF with the chicken Assay Kit (Cayman Chemical Co., Ann Arbor, MI, USA). The interassay and intraassay coefficients of variation were 4.3% and 6.1% for TNF-a, 3.6%, and 6.8% for IL-6, 4.9%, and 7.2% for IL-8, and 5.5% and 8.1% for VEGF.

### 2.3. Molecular analyses

For this purpose, Western blot analyses were done as defined previously [17]. Briefly, samples were homogenized at 1:10 (w/v) in 10 mM Tris-HCl buffer at pH 7.4 containing 0.1 mM NaCl, 0.1 mM phenylmethylsulfonyl fluoride, and 5 mM soluble soybean powder (Sigma, St. Louis, MO, USA.) as a trypsin inhibitor. Samples were centrifuged (15,000 ´ *g* at 4 °C for 30 min), and supernatants were obtained. Protein was separated by 10% SDS-PAGE and transferred onto nitrocellulose membranes. TNF-α, IL-6, IL-8, VEGF, p-IRS-1, p-AKT, p-GSK3-α (Ser21), and p-GSK3-β (Ser9) antibodies (Abcam, Cambridge, UK) were diluted at 1:1000 in the buffer containing 0.05% Tween-20 and used in this study. The membranes were incubated with the primary antibody at 4 °C overnight and subsequently incubated with horseradish peroxidase-conjugated goat antimouse IgG (Abcam, Cambridge, UK). Protein loading was confirmed using an anti-b-actin antibody (Sigma, St. Louis, MO, USA). Protein levels were measured densitometrically using the image analysis software program Image J (National Institutes of Health).

### 2.4. Statistical analysis

All data were shown as the mean ± standard error of the mean. Data were evaluated by analysis of variance using the General Linear Model with the SAS program (SAS Institute Inc.). When a significant F statistic (p < 0.05) was noted, the least-squares mean procedure was performed to differentiate between tools that were significantly different (p < 0.05).

## 3. Results

### 3.1. Effects of genistein on serum inflammatory biomarkers

**Figure 1:**
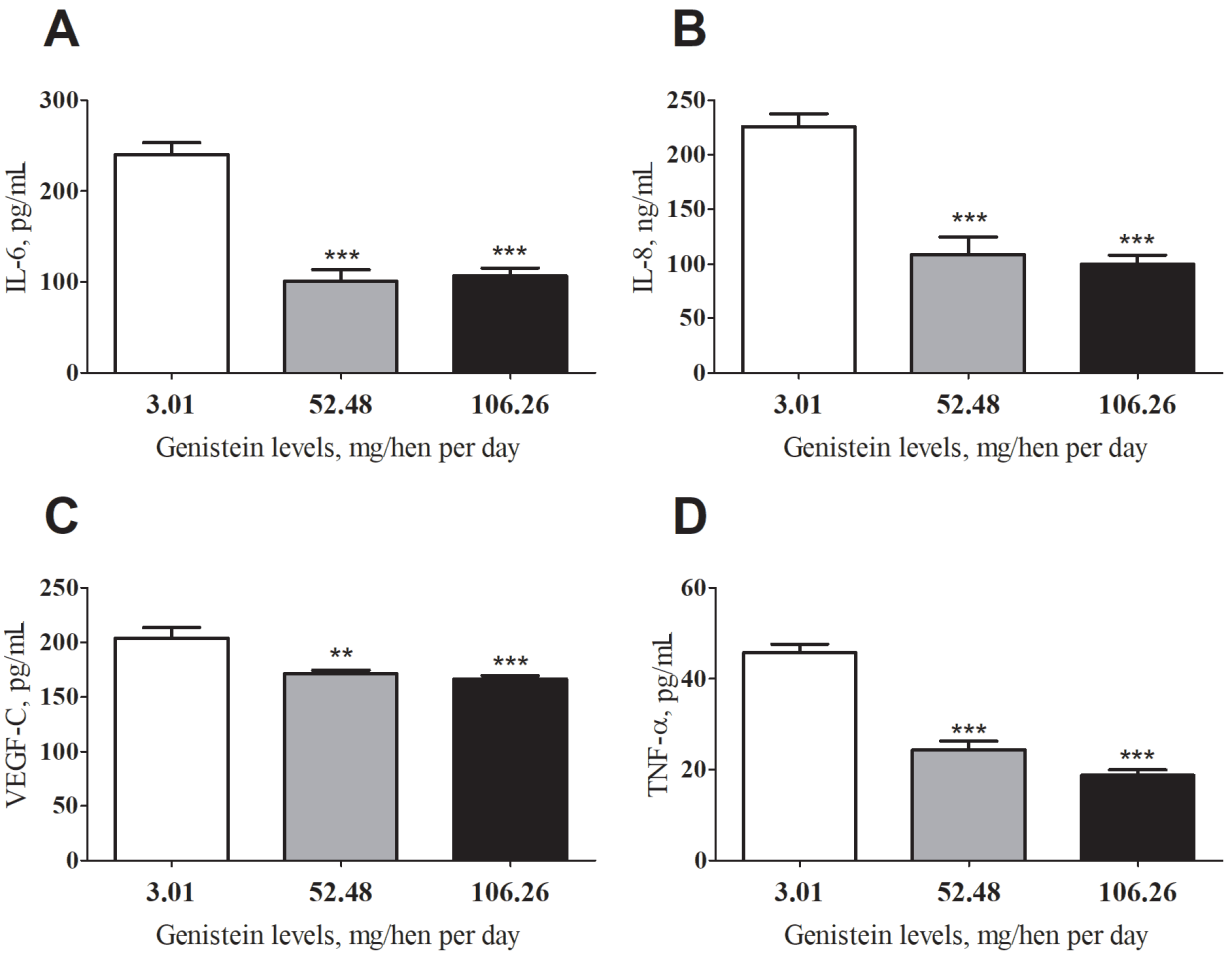
Effects of genistein supplementation on IL-6 (A), IL-8 (B), VEGF-C (C), and TNF- α (D) serum protein levels. *p < 0.05, **p < 0.01 statistically different compared with the control group. TNF-α: tumor necrosis factor-alpha; IL-6: interleukin 6; IL-8: interleukin 8; VEGF-C: vascular endothelial growth factor-C.

Genistein supplementation (52.48 mg and 106.26 mg) significantly decreased serum IL-6 (Figure 1A), IL-8 (Figure 1B), VEGF (Figure 1C), and TNF-α (Figure 1C) levels compared with the control group (p < 0.001). The decrease in IL-8, VEGF, and TNF-α was dose-dependent. The moderate dose of genistein (52.48 mg) was more efficient in decreasing IL-6 level, while the highest dose (106.26 mg) exhibited the highest effect on IL-8, VEGF, and TNF-α (Figures 1A–1D).

### 3.2. Effects of genistein on protein expression levels in ovarian samples

**Figure 2:**
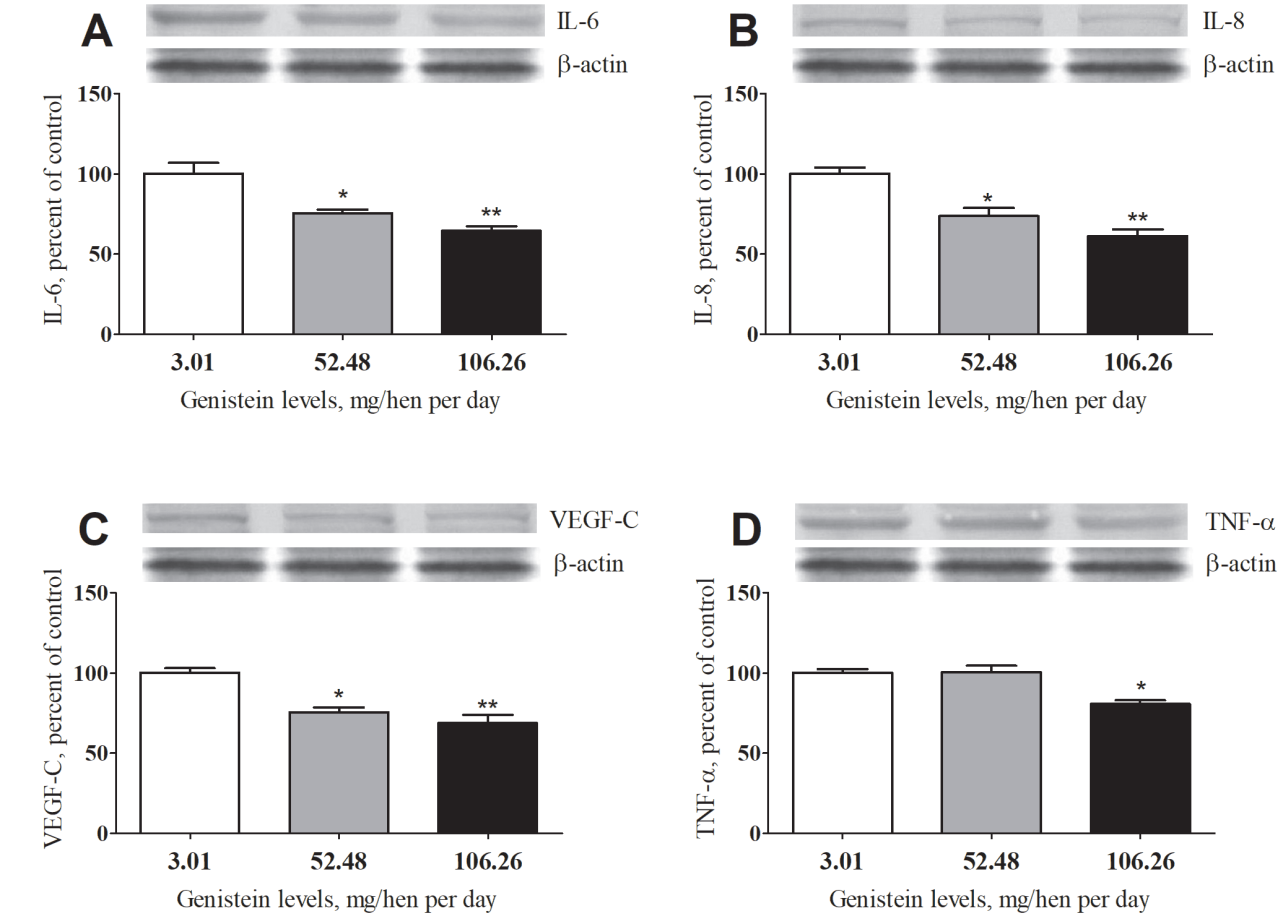
Effects of genistein supplementation on IL-6 (A), IL-8 (B), VEGF-C (C) and TNF-α (D) protein expression levels in the ovarian samples. The intensity of the bands shown was quantified by densitometric analysis. The bar represents the standard error of the mean. Blots were repeated at least 3 times (n = 3) and a representative blot is shown. β-actin was included to ensure equal protein loading. *p < 0.05, **p < 0.01 statistically different compared with the control group. TNF-α: tumor necrosis factor-alpha; IL-6: interleukin 6; IL-8: interleukin 8; VEGF-C: vascular endothelial growth factor-C.

Both moderate (52.48 mg) and high (106.26 mg) genistein supplementation significantly reduced the protein expression levels of IL-6, IL-8, VEGF, and TNF-α in the ovarian samples, indicating that genistein mediates its antiinflammation effects on ovarian cancer through an inflammation signaling pathway. Genistein acts in a dose-dependent manner (Figures 2A–2D).

**Figure 3:**
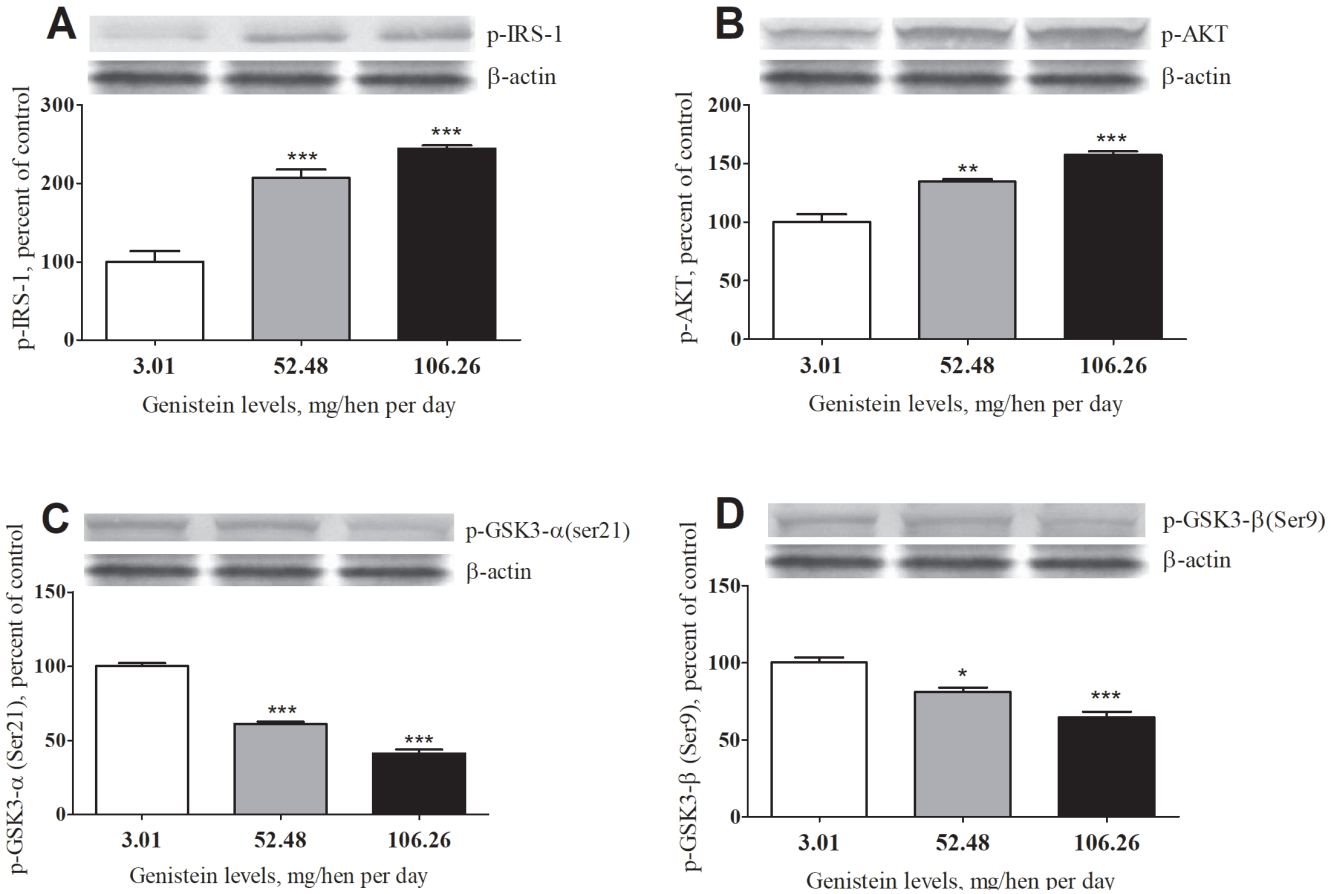
Effects of genistein supplementation on the expression of the phosphorylated protein p-IRS-1 (A), p-AKT (B), GSK-3α (C), and GSK-3β (D) levels in the ovarian samples. The bar represents the standard error of the mean. Blots were repeated at least 3 times (n = 3) and a representative blot is shown. β-actin was included to ensure equal protein loading. *p < 0.05, **p < 0.01, ***p < 0.001 statistically different compared with the control group. IRS-1: insulin receptor substrate 1; AKT: protein kinase B; GSK-3α and β: glycogen synthase kinase-3 alpha and beta.

As illustrated in Figures 3Aand 3B, a significant increase in insulin receptor substrate-1 (p-IRS-1) and p-AKT levels were observed in the genistein-treated groups, with a remarkable effect recorded in animals administered with the highest dose (106.26 mg). On the contrary, genistein supplementation induced a significant reduction in the protein of GSK-3α and β levels compared with the control group (Figures 3C and 3D).

## 4. Discussion

Different experimental and clinical studies propose a preventive role of genistein on several cancers such as colon [20], liver [21], thyroid [22], prostate [23], breast [24], and ovarian cancers [10]. In some patients, advanced therapy of ovarian cancer is related to drug resistance, increased risk of disease recurrence, poor outcome, and severe side effects [10]. Therefore, further research is needed for natural compounds with fewer side effects and easy availability. Frequent consumption of soy products has been recognized to decrease the risks of developing ovarian cancer, mainly due to the high content in an isoflavone called genistein [10]. In the current study, we assessed the properties of genistein on inflammatory biomarkers and glycogen synthase kinase-3 signaling pathway in the ovaries of old laying hens with ovarian cancer. Laying hens, which have been shown to have similar features with humans, develop spontaneous ovarian cancer at a high rate, supplying a favorable experimental model for human ovarian cancer [17]. Indeed, laying hens have shown that ovarian cancer pathogenesis is related to ovulation-induced DNA damage in ovarian cells, similar to humans [25]. Ovarian tumors of laying hens have been classified into four subtypes in terms of tumor stage and grade, including mucinous, clear cell, and serous carcinomas, as those of humans [26]. Recently, the same experimental model has been used by our research team [17].

In the present study, genistein supplementation significantly lowered TNF-α, IL-6, and IL-8 levels in serum compared with the control animals. It has been demonstrated that tumor initiation and progression in the ovary is linked to chronic inflammation activated by oxidative stress [27]. Indeed, tumor cell proliferation and migration stimulate inflammatory cytokine production, including IL-6, IL-8, and TNF-α [28]. The reduction of inflammation biomarkers observed in the current study indicates an antiinflammatory property of genistein in the ovary of old laying hens. This result is similar to that reported by Li and Zhang [29] and Spagnuolo et al. [30], who demonstrated that genistein could suppress inflammatory mediators such as IL-6, IL-8, and TNF-α. Because of the importance of inflammation on cancer occurrence and progression, genistein could be a potential therapeutic agent for ovarian cancer treatment. 

The vascular endothelial growth factor (VEGF) is a member of proteins, performing many endothelial cell functions, such as lymphangiogenesis [31]. VEGF-C is the main factor supporting the metastasis of cancer by activating the VEGF-C/VEGF-R3 signaling pathway and increasing cell mobility and invasiveness [18]. Because VEGF-C signaling affects tumor cells directly, the treatment of ovarian cancer is also mediated by decreasing VEGF-C levels. In the present study, genistein supplementation induced a significant decrease in VEGF-C expression level after treatment. This result corroborates with the previous finding reported by Hu et al. [32], who demonstrated that genistein reduced the expressions of VEGF-C levels in the synovial tissue. The importance of VEGF-C level reduction and the mediation of the treatment via the VEGF-C statements would be controversial, considering the main effects of bevacizumab on the VEGF-A levels rather than VEGF-C levels. Both the paclitaxel and bevacizumab could induce VEGF-C expression, which could be related to tumoral escape [33,34]. Moreover, the anticancer property of genistein observed in the current study is similar to that reported previously by our research group with curcumin in a spontaneously developing hen ovarian cancer model [17].

A possible molecular target for cancer treatment is GSK-3, an extremely conserved serine/threonine kinase [35]. The hyperactivation of GSK-3 may function as an oncogene in different types of cancers, including colon cancer [36], and ovarian cancer [37]. On the contrary, previous reports indicate that GSK-3 inhibitors are capable to suppress the proliferation of malignant cells [38]. Since protein kinase B (PKB or AKT) is a key regulator of protein translation, transcription, cell proliferation, and apoptosis, it is also considered a possible target for cancer prevention/treatment [39]. The activity of GSK-3β is decreased by the phosphorylation of Ser9 and several studies have shown that Ser9 in GSK-3β is phosphorylated and inactivated by AKT [40]. IRS signals proteins that act as intermediates of stimulated cell surface receptors, most remarkably for the insulin and insulin-like growth factor receptors [41]. It has been reported that IRS proteins interface with numerous signaling pathways usually associated with tumor progress and progression by impacting cell metabolism, motility, survival, and proliferation [41]. In cultured bovine adrenal chromaffin cells, Sugano et al. [42] presented that the rise of Ser9-phosphorylation of GSK-3β was followed by reducing IRS-1 and IRS-2 levels. The significant increase in p-IRS-1 and p-AKT expression levels after treatment with genistein may justify the low level of GSK-3 expression. Because the expression of GSK-3 is significantly higher in ovarian carcinoma tissues [43], the significant reduction in GSK-3α and β expression in the current study after treatment with genistein indicates an anticancer effect.

In conclusion, the data presented demonstrate that genistein supplementation exhibited an anticancer effect by reducing inflammatory biomarkers and modulating GSK-3 signaling pathway in the ovaries of laying hens. Genistein is a potential candidate in the chemoprevention and/or treatment of ovarian cancer.
